# Do We Need New Personalized Emergency Telehealth Solutions? A Survey of 100 Emergency Department Patients and a First Report of the Swiss Limmex Emergency Wristwatch: An Original Study

**DOI:** 10.1155/2012/736264

**Published:** 2012-08-16

**Authors:** Malek Tabbara, Thomas Hodel, Urs Müller, Gabi Briner, Heinz Zimmermann, Aristomenis K. Exadaktylos

**Affiliations:** ^1^Department of Emergency Medicine, Inselspital, Bern University Hospital, CH-3010 Berne, Switzerland; ^2^Health Care Research Institute AG, CH-8005 Zürich, Switzerland

## Abstract

Development of new personal mobile and wireless devices for healthcare has become essential due to our aging population characterized by constant rise in chronic diseases that consequently require a complex treatment and close monitoring. Personal telehealth devices allow patients to adequately receive their appropriate treatment, followup with their doctors, and report any emergency without the need of the presence of any caregivers with them thus increasing their quality of life in a cost-effective fashion. This paper includes a brief overview of personal telehealth systems, a survey of 100 consecutive ED patients aged >65 years, and introduces “Limmex” a new GSM based technology packaged in a wristwatch. Limmex can by a push of a button initiate multiple emergency call and establish mobile communication between the patient and a preselected person, institution, or a search and rescue service. To the best of our knowledge, Limmex is the first of its kind worldwide.

## 1. Background

Patients with complex comorbidities who receive traditional healthcare services fail to show long-term compliance with prescribed regimens, medications, and dietary restrictions [1]. It has been shown, however, that strategies that include extrinsic motivators promote long-term compliance and reduce recidivism [2]. As the population is aging, chronic disease requiring complex treatment and close monitoring is increasing. Moreover, the number of medical personnel is dropping and both homecare nursing and medical staff are expensive. It has therefore become necessary to search for ambitious alternative solutions, which could help the patient to receive adequate treatment and followup from their doctors and to report any emergency, even in the absence of any caregivers and without having to memorize or save emergency numbers, especially when the primary contact is not available. In an ongoing pilot study at our university hospital emergency department, 40% of interviewed patients (>65 years) were not able to recall emergency telephone numbers. Forty-two percent (42%) of them did not own a mobile phone. Thirty-three percent (33%) thought that, in an emergency, more than one hour might pass until somebody noticed. Thirty-seven patients (37%) thought that up to 30 minutes might pass before anyone was notified of their emergency. Almost 40% of the patients communicated their wish to have faster access to medical assistance. Telemedicine and telehealth, especially personalized handheld devices, can offer a new approach to these problems. 

Telemedicine (TM) is the use of medical expertise, medical equipment, computer hardware and software, telecommunication infrastructure and the Internet as a healthcare system [3]. Telehealth (TH) is the delivery of health-related services and information via telecommunication technologies. This is an extension of telemedicine and encompasses preventive, promotional, and curative aspects. TH involves the use of the tools to produce, transmit, manage, and share digitalized information and comprises the use of applications that allow medical activities, including teleconsultation, medical telemonitoring, and medical tele-assistance, as well as remote monitoring and data devices. The latter are known as televigilance or teledata [4]. The first TH system, operating over standard telephone lines, was used for the remote diagnosis and treatment of patients requiring cardiac resuscitation and was developed and marketed by MedPhone Corporation in 1989 [5]. 

Advances in the area of mobile and wireless communication for healthcare, along with the improvements in information science, have allowed the design and development of new personalized healthcare services, increasing the patient's independence and improving his ability to control and manage his life [6, 7]. Moreover, many studies have shown that TH can improve the quality of life and clinical outcome and is a cost-effective tool. Noel et al. studied 104 patients with complex heart failure, chronic lung disease, and/or diabetes mellitus who were equipped with a TH device functioning on their home landline. Their twelve-month observational study proved that TH used significantly fewer resources and improved cognitive status, treatment compliance, and the stability of the chronic disease of the homebound elderly with common complex comorbidities [8]. The UK Department of Health's Whole System Demonstrator (WSD)program was launched in May 2008 and is the largest randomized control trial of TH in the world, involving 6191 patients and 238 general practitioners. Three thousand and thirty people (3030) with one of three conditions (diabetes,chronic heart failure, ordisease) were included in this trial. The trial showed a 45% reduction in mortality rates, a 20% reduction in emergency admissions, a 14% reduction in elective admissions, a 14% reduction in days in hospital, and an 8% reduction in tariff costs [9]. Similarly, in a systematic review evaluating nine randomized clinical trials on heart failure (967 patients), Dang et al. noted that six studies found a 27%–40% reduction in overall admissions; two studies demonstrated a 40%–46% reduction in heart failure-related admissions; three studies found a significant reduction in mortality (30%–67%); three studies showed significant reduction in healthcare utilization costs; two studies also found a 53%–62% reduction in bed days of care; two studies showed a significant reduction in the number of emergency visits; four studies demonstrated significant overall improvement in outcomes with the use of telemonitoring [10]. TH could also become a very effective tool in providing guidance and support in situations of loss of autonomy, by equipping users with devices that will provide monitoring, continuity of treatment, and reinforcement of social ties. Under no circumstances is TH meant to replace a doctor's presence with a computerized tool but was rather designed to meet the needs and expectations of patients more effectively—people who are vulnerable, dependent, and/or disabled. This paper aim to present (1) an overview of existing technologies, (2) a survey of 100 consecutive ED patients aged >65 years, and (3) the Swiss Limmex emergency wristwatch.

## 2. Mobile and “Handheld” Telemergency ****Devices—Reality or Fiction?

All TH personal devices—including watch-based glucose and ECG monitoring devices—have been used experimentally and are still not commercially available. The main drawback remains the complexity of the devices that are being tested. The problem could lie in the device itself or in the communication technology that is being used. In the hope of detecting out-of-hospital cardiac arrest, Rickard et al. studied the ability of Wriskwatch, a watch-based pulse detection that could notify emergency services of a pulseless state. It was unclear how sensitive or specific this study was in detecting true out-of-hospital cardiac arrest. The authors were also unable to determine whether the device can activate emergency medical systems, and, if so, whether this would improve response times and patient outcomes [11]. 

TeleAlarm S12 and The Red Cross Emergency equipment (Table 1) consist of an alarm button and a speaker phone with microphone and speakers. The alarm button can be worn on the wrist or around the neck. These systems are being currently used in Switzerland and serve the same purpose to our proposed device “Limmex” with some important differences in some key aspects. The major drawback of these systems is that the watch (alarm button) must be connected to a base station within a range of 50 meters and that this base station must use a standard active phone line (landline) as a mode of communication and needs an electrical power supply. Thus, the watch can only be used within a 50 meters indoor perimeter and no outdoors activities are possible [12]. 

In addition, these systems are expensive, costing about 250 US dollars for a two month lease, about 600 US dollars for the device, and between 40–60 US dollars monthly rent on a long-term contract. That does not include the price of any technical adjustments (electricians), call charges and the telephone line. They can be complicated to install—in contrast to a plug and play devices—and, above all, these devices somehow label and stigmatize the user as “sick” due to their bulkiness and indiscreet size. Therefore, a simple, reliable, and easy-to-wear device is needed to be able to trigger an emergency call wherever and whenever the patient is in need. 

## 3. Access to Emergency Numbers: A Survey

 In an attempt to study the possible role and impact of telemedicine handheld devices on the elderly population, we designed a questionnaire (see Supplementary Material available online at doi:10.1155/2012/736264) and asked 100 consecutive patients older than 65 years who visited our emergency room to complete it. The questionnaire included questions about patients demographics, level of education, reason for admission, emergency numbers memorized by the patient, access to a mobile phone, time needed for the patient to be noticed by someone in case of an accident at home, the need for a device to facilitate call for help in case of an emergency and their willingness to buy such a device. Fifty-seven percent (57/100) of the participants in this survey were males. The mean age of the patients was 75 years. 25% (25/100) held a higher professional degree or a university degree, 48% had professional training, and the remaining 27% had not completed any education after compulsory school. When asked about their living situation, 29% stated that they lived alone, while 71% lived with at least one other person in the household. Thirty-seven percent (37%) thought that in case of an emergency, if they were unable to speak, another person would notice their condition within half an hour or less, 21% answered that it would take between half an hour to an hour for someone to notice them, and 33% stated that it could take more than 1 hour to be noticed. The remaining 9% did not answer this question.

 Eighty percent (80%) of the patients stored the phone number of their family doctor, while 56% of patients had this number on their mobile phone or written on a card in their wallet; only 24% knew it by heart. Fifty-eight (58%) stated that they used a mobile phone while 42% did not. More than 60% (63/100) knew the phone number of the ambulance and approximately 25% (23/100) could recall the number for Swiss Air Rescue. In addition, only 49% (49/100) of the interviewed patients knew the emergency numbers of the police and the fire department. 

 When asked if they would worry that no one would notice them in case of a medical emergency, 57% denied any worry while 43% expressed this fear. Thirty-seven percent (37%) wished for a more rapid method to alarm a helpful person or an emergency service. Approximately 60% (58/100) of those interviewed noted they would feel safer if they had a device which enabled them to alarm a close person of their choice, the family doctor, or an emergency service in any medical emergency. Seventy-five percent (75%) of participants declared they were prepared to spend 500 Swiss francs (~600 US dollars) for a telemergency device, while 25% agreed to spend 1000 Swiss francs or more if they thought that their situation had worsened to a point that required such device.

The numbers on this survey and the lack of a reliable and easy-to-wear device that enables patients to trigger an emergency call indicate the need for a groundbreaking device that facilitates communication between patients and-emergency services. 

## 4. Limmex Telemergency Watch from Switzerland: Just a New “Gadget”—or a True Help in Emergency Situations?

**Figure 1:**
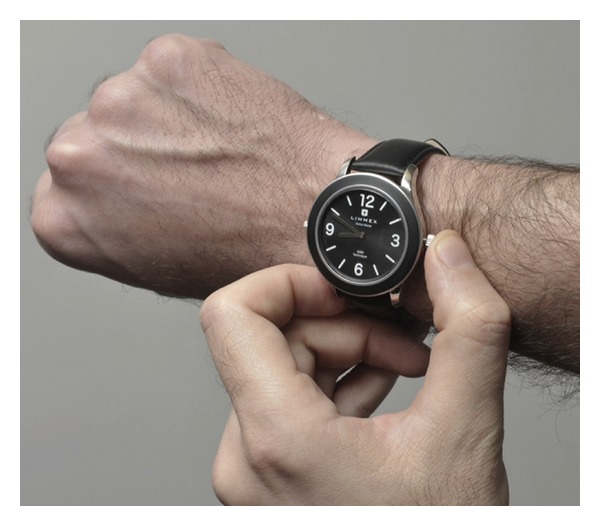
Limmex emergency wrist watch: there are two crowns, the one on the left for adjusting time and the one on the right (the larger one) for triggering the emergency call.

**Figure 2:**
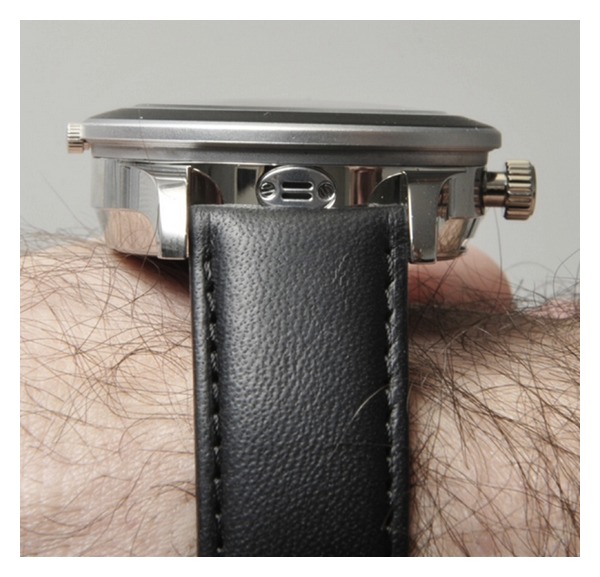
Limmex emergency wrist watch: wrist shot showing the dimensions of the watch and the microphone.

The Swiss company“Limmex AG” and the Centre Suisse d' Electronique et de Microtechnique (Swiss Center for Electronics and Microtechnology) have jointly developed “Limmex” a new emergency watch (Figures 1 and 2). CSEM SA, founded in 1984, is a private research and development center specializing in microtechnology, nanotechnology, microelectronics, system engineering, and communications technologies [12, 13]. Together with the Department of Emergency Medicine, Bern University Hospital [14], several clinical applications were tested and the watch was further developed. The University Hospital Emergency Department is a tertiary unit and one of the largest in Switzerland.

The Limmex emergency watch features a Swiss-made Ronda Quartz movement. It is waterproof against splashes. It has a lithium-polymer rechargeable battery. If there is no emergency, the battery should be recharged every few months. For security reasons, Limmex suggests recharging the battery after an emergency call. The watch integrates a cloud-based technology, allowing remote configuration of settings. Limmex watch costs about 400 US dollars and the monthly contract charges are 25–35 US dollars. There are no hidden extracosts for technical implementation.

**Figure 3:**
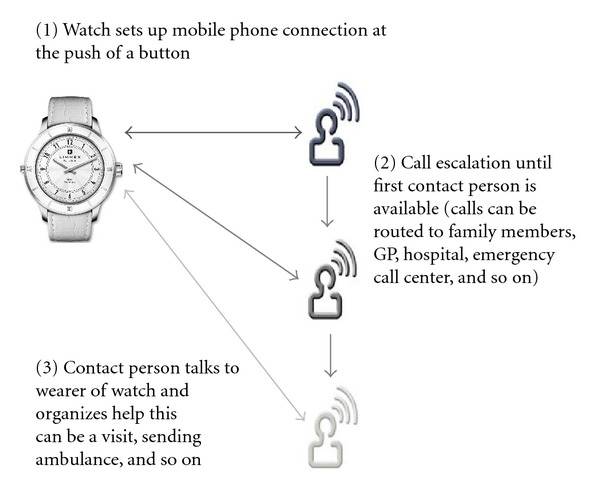
Limmex Emergency Watch—escalating alarm mechanism.

The uniqueness of the Limmex is that the watch enables the wearer to request assistance at the push of a button and to speak with preselected persons or services (e.g., police, hospital, ambulance, etc.). Unlike other TH personal devices, the Limmex watch is based on GSM mobile technology; it contains an irreplaceable SIM card on which the user can save up to ten phone numbers. The diagram (Figure 3) is self-explanatory and summarizes how the Limmex system works. The patient wearing the watch can triggeran emergency call by pressingand releasing the emergency callbutton; a beep then sounds for 15 seconds. During these15 seconds,he can stopthe alarm by pressingthe buttonagain. If he does not press it again during these 15 seconds, the Limmex watch will call thefirstrecipienton his preset list; this could be a family member, his family doctor, an emergency center, or an ambulance service. The recipient is immediately prompted with the following message: “This isan emergency callLimmex. Pleasepress5to respond!” If the recipientpresses key5 on the telephone, aconnectionis establishedwith the patient. If thefirst recipientdoes not press key “5” (or ifthe line is busyor an answering machineanswers the call), the following numbersare calledone after the other untila person responds tothe emergency callby pressing key “5.” If an emergency call is triggered, the watchcanbe calledfor 30minutes to ensure that help has beenorganized. Duringthis time, the watchwillautomatically accept incoming calls [13].

 The watch currently works all over Switzerland. The potential applications of this emergency watch are numerous and are being investigated by our team. In addition to elderly patients with complex comorbidities, this innovative emergency watch can also increase the quality of life in individuals with illnesses such as epilepsy, cardiac problems or severe allergies, pregnant women, or adolescents with juvenile diabetes—any of whom might find themselves in an emergency situation and need immediate assistance. We are currently testing the watch in several patient groups. The use of Limmex can also have a positive repercussion on the healthcare system itself; patients who would have been hospitalized for a longer time for surveillance or for social reasons would be able to be discharged home with a hospital-sponsored Limmex for a limited time; this would allow the patients to call for help at all times once at home and might enhance their quality of life and free hospital beds. The Limmex watch can be used outside or even inside just anywhere where you have wireless coverage, thus the wearer is able to count on a reliable system which operates outside his own four walls, is discreet, and which requires no special installation. In addition, Limmex provide its users with a wide selection of styles for men and women and not just a standard model such as the tele-alarm. 

## 5. Summary 

The Limmex emergency wristwatch can play an important role in the TH system by providing an Alert-Diagnose-Treat service to the patient/person wearing the device and might even save his life. This applies particularly to patients living alone. Furthermore, Limmex could be used for communication between the patient and the caregiver (whether he/she is a family member, a nurse or a physician), in which case the patient can report that he is doing well, or that he has taken the specific treatment at the scheduled time. In contrast to a mobile phone, Limmex is instantly accessible at all times. At the push of a button, an emergency call can be initiated and help arranged. A future prospective study will be designed to look into the social and economic impact of using such device.

## Supplementary Material

The questionnaire included questions about patients demographics, level of education, reason for admission, emergency numbers memorized by the patient, access to a mobile phone, time needed for the patient to be noticed by someone in case of an accident at home, the need for a device to facilitate call for help in case of an emergency and their willingness to buy such a device.

## Figures and Tables

**Table 1:** Comparison between telealarm/red cross watch and Limmex watch.

	Telealarm/red cross watch	Limmex watch
Technology	Landline	GSM
Ability to use outdoor	No	Yes
Stigmatize patient	Yes	No
Cost	+++	++
Power supply needed	Yes	No
Rechargeable battery	No	Yes
Waterproof	No	Yes
