# Supplementary material for: Do We Need New Personalized Emergency Telehealth Solutions? A Survey of 100 Emergency Department Patients and a First Report of the Swiss Limmex Emergency Wristwatch: An Original Study
Source: Int J Telemed Appl. 2012 Aug 16;2012:736264. doi: 10.1155/2012/736264 (PMC3431086; doi:10.1155/2012/736264)
Supplement: Supplementary file 1 — The questionnaire included questions about patients demographics, level of education, reason for admission, emergency numbers memorized by the patient, access to a mobile phone, time needed for the patient to be noticed by someone in case of an accident at home, the need for a device to facilitate call for help in case of an emergency and their willingness to buy such a device. [file 736264.f1.pdf]

Appendix A

Notfallzentrum

Anonymous Patient Questionnaire

Theme: Access to Emergency Numbers

Gender: ☐ male ☐ female

Nationality: ☐ CH ☐ Other

Age: ________________

Your highest completed level of education:

☐ None
☐ Compulsory school (primary, technical college, secondary school, district school, orientation school, grammar school, special needs school)
☐ Completed professional training
☐ Matura [equivalent to A-Levels]
☐ Higher Professional Training
☐ University

Reason for admission: ☐ Disease ☐ Accident

1. Which emergency numbers do you know?
   - ☐ GP
   - ☐ Ambulance
   - ☐ Air rescue
   - ☐ Emergency centre/ Hospital
   - ☐ Other emergency number
