# Supplementary material for: Do We Need New Personalized Emergency Telehealth Solutions? A Survey of 100 Emergency Department Patients and a First Report of the Swiss Limmex Emergency Wristwatch: An Original Study
Source: Int J Telemed Appl. 2012 Aug 16;2012:736264. doi: 10.1155/2012/736264 (PMC3431086; doi:10.1155/2012/736264)
Supplement: Supplementary file 2 [file 736264.f2.pdf]

Appendix A

2. How do you contact the emergency centre in the event of an emergency?
   - ☐ I phone them up myself.
   - ☐ My partner, friend or relative phones them.
   - ☐ My GP makes an appointment.
   - ☐ Nursing service or other nurses phone them.
   - ☐ I just turned up. / I was brought there.
   - ☐ Other, namely___________________________

3. Do you use a mobile phone?
   - ☐ Yes
   - ☐ No

4. How many people do you live with?
   - ☐ I live alone.
   - ☐ Together with my partner or spouse/friends/brothers or sisters/parents

5. Where do you live?
   - ☐ At home
   - ☐ Protected accommodation
   - ☐ Nursing home
   - ☐ Other, namely___________________________

6. If something happens to you at home and you cannot speak or telephone yourself: How long do you think you would usually have to wait before anyone notices that something has happened to you?
   - ☐ < 5 minutes
   - ☐ 5 - 15 minutes
   - ☐ 16 - 30 minutes
   - ☐ 31 - 60 minutes
   - ☐ 61 - 120 minutes
   - ☐ More than 120 minutes
   - ☐ No-one would notice.
   - ☐ I don’t know.
