# Supplementary material for: Do We Need New Personalized Emergency Telehealth Solutions? A Survey of 100 Emergency Department Patients and a First Report of the Swiss Limmex Emergency Wristwatch: An Original Study
Source: Int J Telemed Appl. 2012 Aug 16;2012:736264. doi: 10.1155/2012/736264 (PMC3431086; doi:10.1155/2012/736264)
Supplement: Supplementary file 3 [file 736264.f3.pdf]

Appendix A

Now a few final questions (for all patients)

1. Do you worry that something might happen to you and non-one would notice?
   - ☐ no
   - ☐ sometimes
   - ☐ often
   - ☐ always

2. Do you sometimes wish that you could contact someone to help or the emergency centre more rapidly?
   - ☐ no
   - ☐ yes

3. Could a device – such as a clock – give you additional security, if you could press a button on it at any time to contact someone to help, your GP or the emergency centre?
   - ☐ no
   - ☐ probably not
   - ☐ probably yes
   - ☐ yes

4. If you had 1000 francs spare for a device of this sort how much would you be willing to pay?
   - ☐ 0
   - ☐ 100
   - ☐ 200
   - ☐ 300
   - ☐ 400
   - ☐ 500
   - ☐ 600
   - ☐ 700
   - ☐ 800
   - ☐ 900
   - ☐ 1000

Other comments or suggestions:

____

____

____

Many thanks for your assistance!
